# Stroke Following Blunt Head Trauma: A Case Report and Review of the Literature

**DOI:** 10.7759/cureus.77147

**Published:** 2025-01-08

**Authors:** Judah Stiefel, Mollie Schear, Comfort Anim-Koranteng, Chibuzo Ukonu, Salama Fawzy

**Affiliations:** 1 Internal Medicine, Touro College of Osteopathic Medicine, New York, USA; 2 Internal Medicine, NYC Health + Hospitals/Harlem, New York, USA; 3 Psychiatry, NYC Health + Hospitals/Harlem, New York, USA; 4 Neurology, NYC Health + Hospitals/Harlem, New York, USA

**Keywords:** brain ischemia, computed tomography angiogram (cta), endothelial injury, intracranial thrombosis, lower limb numbness, neck massage, stroke

## Abstract

Ischemic stroke due to blunt traumatic cerebrovascular injury (TCVI) is rare and often overlooked, particularly in patients without any typical predisposing factors. Approximately 1-2% of patients with blunt cerebrovascular trauma experience extracranial injuries, and around 10% of these patients develop strokes, resulting in an estimated 4,800 cases annually in the United States. These strokes can present with atypical symptoms, causing a delay in diagnosis. We report a case of a 36-year-old female who experienced headaches, left-sided weakness, and numbness following a football-related injury, and frequent use of a neck massager. Initially, her symptoms were attributed to a complex migraine; however, an MRI revealed a right thalamic infarct caused by the occlusion of the right posterior cerebral artery (PCA). Subsequent imaging showed further ischemic changes.

As the patient's condition progressed, her management required a change to include heparin after initial treatments with aspirin and clopidogrel proved ineffective. This report highlights the challenges in diagnosing TCVI-induced stroke due to its uncommon presentation, atypical symptoms, and low clinical suspicion, especially in young adults. It underscores the importance of considering ischemic stroke in patients who have experienced neck trauma and raises awareness about the potential risks of unregulated neck massagers, which may cause vascular injury. Early cerebrovascular imaging, such as CT angiography (CTA), should be considered for trauma patients who present with neurological symptoms to facilitate timely diagnosis and treatment.

## Introduction

Ischemic stroke due to blunt traumatic cerebrovascular injury (TCVI) resulting in hyperextension or hyperflexion of the neck is uncommon. It is associated with a low index of suspicion in patients, especially those without pertinent predisposing factors for stroke. Approximately 1-2% of patients admitted after blunt cerebrovascular trauma have extracranial injury [1,2]. Of the patients reporting TCVI, roughly 10% are found to have strokes, accounting for 4,800 TCVI strokes in the United States reported each year [3,4]. However, they may have atypical symptoms during initial evaluation and diagnosis [5].

Therefore, it is important to have a high index of suspicion for stroke in patients with TCVI for prompt diagnosis and management. We discuss a case of a 36-year-old female who presented initially with indeterminate neurological symptomatology suggesting complex migraine and was eventually diagnosed with right thalamic and inferior temporal lobe infarct from occlusion of the right posterior cerebral artery (PCA).

## Case presentation

A 36-year-old right-handed female presented to the emergency department with a throbbing headache, new-onset ophthalmalgia, weakness, and numbness of the left upper and lower extremities. She reported being hit by a football in the chin and neck area while playing Australian football with friends six days before the presentation. Examination by medical personnel on the sports field had been normal on the day of trauma. Due to soreness from the ball, she had used the neck massager more frequently than usual. On the day of the presentation, she had used an electronic hand-held neck massager with firm rolling balls about 10 minutes before the onset of symptoms. She denied loss of consciousness, nausea, and vomiting. Her past medical history revealed migraines, of which she was not on treatment. She denied smoking or alcohol intake but endorsed occasional marijuana use but none within the past six months during the review of other potential contributors.

**Figure 1:**
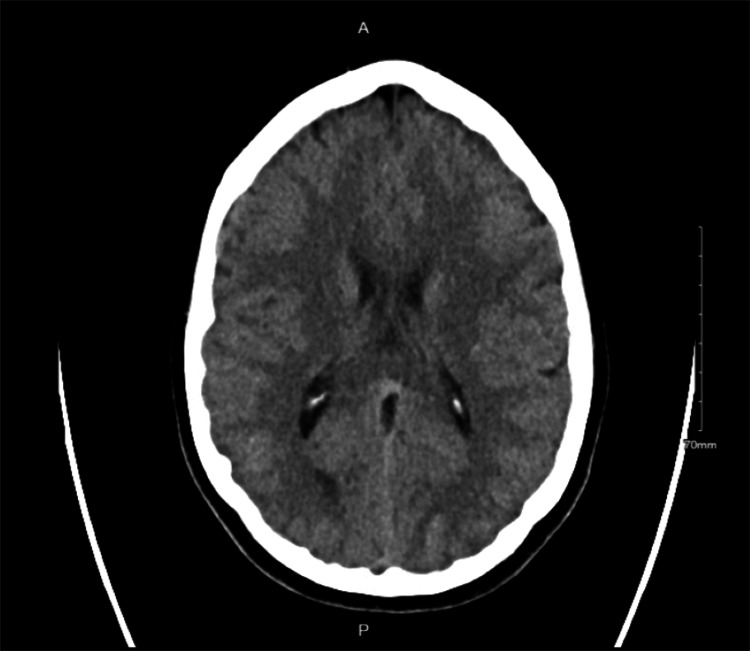
CT scan of the head showing normal findings CT: computed tomography

Her vitals were notable for a temperature of 98.2 °F, a pulse of 86 beats per minute, a blood pressure of 148/88 mmHg, a respiratory rate of 18 breaths per minute, and an oxygen saturation of 100% on room air. There was evidence of total sensory loss to touch and a pinprick of the left side with a National Institutes of Health Stroke Scale (NIHSS) score of 2 as the initial symptoms of ophthalmalgia, left-sided hemiparesis, and headache had resolved by the time of the examination. A head CT scan without contrast (Figure 1) was negative for any acute intracranial pathology.

**Figure 2:**
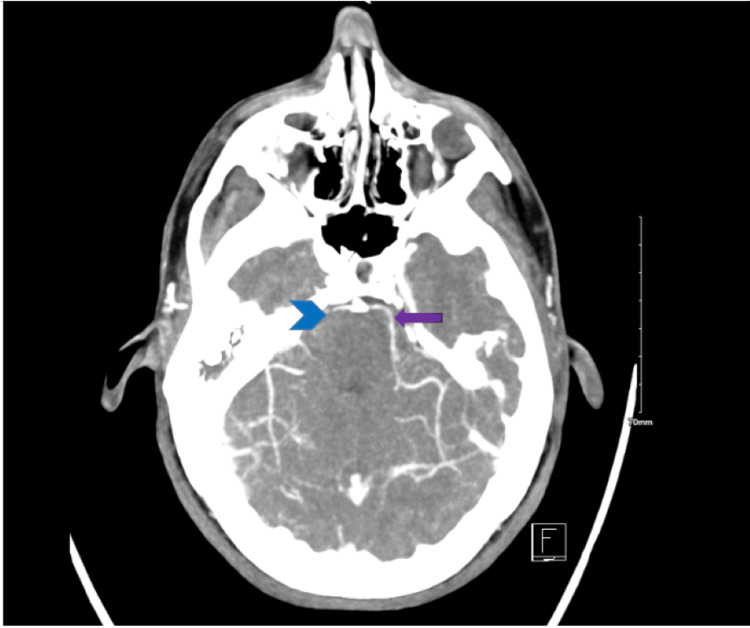
CTA of the head and neck The blue arrowhead shows the loss of filling defect of the right PCA while the purple arrow indicates continued flow of contrast in the left PCA CTA: computed tomography angiography; PCA: posterior cerebral artery

A CTA of the head and neck showed some evidence of significant stenosis (Figure 2).

Two hours after the presentation, the patient had a witnessed general tonic-clonic seizure lasting 30-60 seconds, which was aborted with midazolam in the ER. She was managed on levetiracetam, aspirin, clopidogrel, and atorvastatin as per Stroke protocol. The working differential diagnosis at that time included transient ischemic attack (TIA), stroke, complex migraine, or seizure. She underwent an MRI of the brain due to the persistence of left-sided numbness after 24 hours, which demonstrated an acute right thalamic infarct. A transthoracic echo with bubble study was negative for any intracardiac shunt, and a hypercoagulability panel was normal except for low normal protein C and antithrombin III (ATIlI) (Table 1).

**Table 1:** Hypercoagulable workup results

Test	Patient value	Normal range
Antithrombin III (ATIlI) activity	84%	85-135%
Antithrombin III (ATIlI) antigen	24 mg/dl	19-31 mg/dl
Beta 2 glycoprotein1 antibody	Negative	Negative
Cardiolipin antibiodies	Negative	Negative
Factor V	56%	50-150%
Factor V Leiden case	Normal	Normal
Factor VIII assay	67%	60-125%
Homocysteine	7.3 umol/l	<15 umol/l
Protein C activity	72%	74-150%
Protein C antigen	79%	80-184%
Protein S free antigen	86%	61-131%
Phosphatidyl serine antibodies	IGM (19 units), IGA (<1), IGG (<9)	IGM (0-30), IGA (0-19), IGG (0-30)

**Figure 3:**
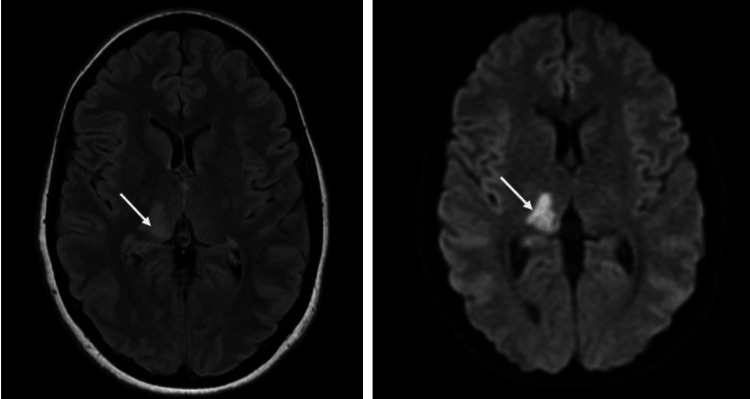
MRI axial view of the brain The left image is the T2/FLAIR, and the right image is the DWI, showing the high signal intensity indicated by arrows DWI: diffusion-weighted imaging; FLAIR: fluid-attenuated inversion recovery; MRI: magnetic resonance imaging

**Figure 4:**
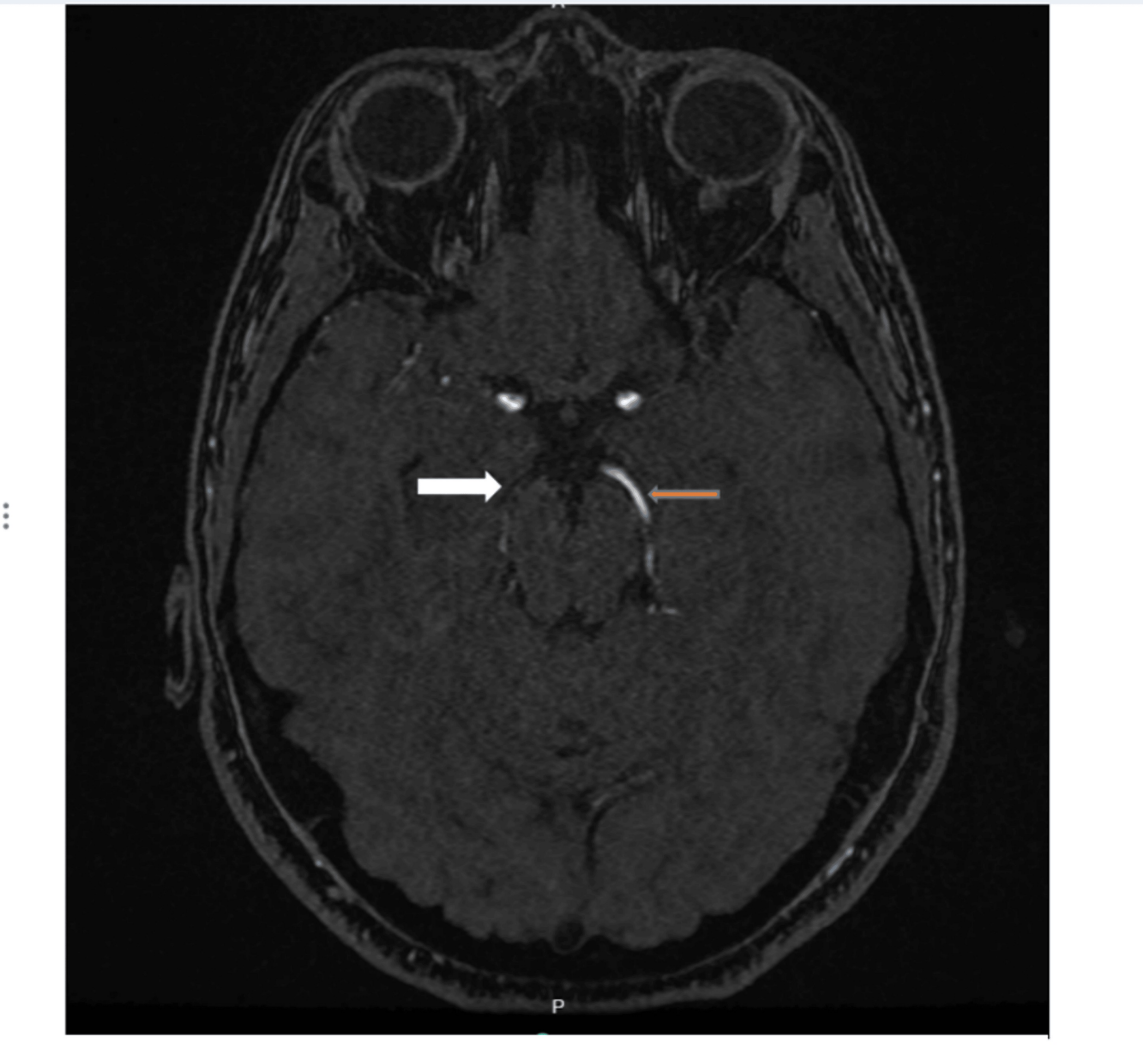
MRA of the head with contrast The image shows occlusion of the right posterior cerebral artery at the level of the distal aspect of the P1 segment (white arrow) with intact left posterior cerebral artery without any stenosis (orange arrow) MRA: magnetic resonance angiography

The patient's symptoms progressed throughout admission within days, with the development of a gross motor deficit, with the power of 2/5 of the left upper and lower extremities, and a shuffling gait. On day seven, a repeat brain MRI and MR angiography (MRA) revealed a new right medial temporal lobe infarct with occlusion of the right PCA at the level of the distal aspect of the P1 segment (Figures 3, 4).

Due to the persistence of acute stroke on aspirin and clopidogrel, management was switched to a heparin drip. There was no recurrence of generalized tonic-clonic seizures during admission; however, the patient did have involuntary movements of the left upper and lower extremities, which was perceived as clonus. A repeat hypercoagulability workup returned normal, and she was discharged on aspirin, clopidogrel, and atorvastatin for stroke management. The patient was discharged to an acute rehabilitation facility to aid in the recovery of left-sided paraparesis. Her discharge medications included aspirin, clopidogrel, atorvastatin, gabapentin, topiramate, oxcarbazepine, and trazodone. Unfortunately, the patient has been lost to follow-up and her recovery progress is unknown.

## Discussion

Diagnosis

This report involves a 36-year-old female who presented to the ER with new-onset ophthalmalgia, left-sided weakness, and numbness in the upper and lower extremities. The patient reported a minor head injury days prior, followed by regular use of a pulsatile electronic neck massager leading up to the onset of symptoms. Initial CT scans were negative, but upon repeat MRI, an infarct of the right thalamus was observed. 

The initial differential diagnosis of the case included atypical migraine presentation, TIA, concussion syndrome, stroke, and seizure. While stroke was initially considered very unlikely based on the NIHSS score of 2, the follow-up MRI confirmed a diagnosis of an ischemic stroke in the region supplied by the right PCA. The location of the stroke, along with the CTA findings, pointed to a lesion of the PCA. Based on the patient's history of frequent use of a neck massager to the back of the neck where the vascular lesion was found, we proposed that the mechanism of injury was a vascular lesion caused by this massaging technique. The fact that her initial symptoms had occurred soon after the use of the massager endorsed this hypothesis.

Strokes are very rarely reported in young adult populations, especially those with no known predisposing factors. The incidence of stroke in patients aged 35-44 years is between 30-120 per 100,000 people yearly compared to 670-970 per 100,000 in those aged 65-74 years [6]. Thus, ischemic stroke is often low on the differential diagnosis in young adults with neurological symptoms after trauma. Atypical symptomatology also can play a role in the delayed diagnosis of TCVI-induced stroke. One study found that of 194 trauma patients, 11 had ischemic strokes. Of them, none were diagnosed with acute ischemic stroke on admission, and it was shown that diagnosis was delayed by an average of 1.8 days following patient presentation. In seven of these 11 patients, the diagnosis of stroke was confirmed with cervicocranial vascular imaging and one with intracranial imaging [6]. In another retrospective study involving 479 TCVI patients, 24 (5%) had a stroke on admission, and 12 had a stroke during their hospitalization. The timing of stroke onset among those who developed it later was about 21 hours [7].

Pathophysiology 

Non-penetrating trauma can damage the cervical, carotid, and vertebral artery wall layers, causing tears in the vascular intima. Intimal tears may be caused by artery elongation from motions such as neck torsion or rapid stretching, exposing underlying subendothelial collagen, activating platelets, and beginning thrombus formation, which embolizes to cause vascular occlusion. In such patients, thrombus formation may be accelerated due to post-traumatic hypercoagulability [8].

Vertebral artery injury with no predisposing factors most commonly results from trauma [9,10]. Intimal tears allow blood to enter the walls of the vasculature, leading to stenosis and occlusion. Additionally, elastic lamellar damage may expand to the adventitia, leading to the development of aneurysms [11]. Trauma specific to the neck may cause injury to any of these vessels [12]. Dissections of the vertebral artery may rarely occur in cases with no history of predisposing disease. In our patient, based on the onset of the stroke soon after the use of the neck massager as well as the location of the ischemic event in the PCA, the use of the neck massager likely played a role in the clot formation and embolization of the clot.

Clinical significance

Diagnosing ischemic stroke may be difficult in trauma patients as confounding injuries may create atypical symptomatology, as is emblematic from our patient's presentation. Additionally, atypical or delayed stroke symptomatology may cause delayed or altogether missed diagnosis of a TCVI stroke. Patients who present with trauma to the back of the neck or related torsion or hyperextension are at increased risk for ischemic stroke. TCVI-induced ischemic stroke may need to be considered in the case of trauma to the back of the neck. However, conventional initial stroke imaging involves CT, which is unlikely to show an ischemic stroke during the acute initial presentation.

Patients presenting with atypical stroke symptoms may receive an initial misdiagnosis and the opportunity for thrombolytic treatment may be missed [13]. In our patient, a history of past migraines and atypical stroke symptoms with an initial NIHSS score of 2 led us to avoid recommending tissue-type plasminogen activator therapy, which may have mitigated her progressive thalamic symptoms. Increased use of CTA in the screening of cerebrovascular trauma patients has led to a growing number of TCVI diagnoses [14]. Early use of CTA may be useful in the diagnosis of stroke in patients who have suffered neck trauma and should be considered as an option during the initial screening.

Concerning electronic massage devices, other cases of injury to the vertebral artery related to pulsatile massage devices have been reported recently [15]. These devices have become more accessible in the consumer market, varying widely in both quality and design. These devices are unregulated, and instructions offer little in the way of caution regarding usage to the neck. With the increasing availability of such products, clinicians need to consider trauma induced by neck massage devices when treating patients with vague neurological symptoms.

## Conclusions

We discussed a case of a 36-year-old woman who presented with headache, ophthalmalgia, left-sided weakness, and numbness after using a neck massager more frequently after being struck by a football in the chin and neck area six days earlier. Initial examination and CT scan were normal, but two hours later, she had a tonic-clonic seizure. MRI revealed an acute right thalamic infarct and further imaging showed a right medial temporal lobe infarct due to occlusion of the right PCA. She was treated with stroke protocol medications, including aspirin, clopidogrel, and atorvastatin. After a progression of motor deficits, management was adjusted to heparin, and she was discharged to rehabilitation with ongoing stroke management. Unfortunately, she was lost to follow-up, and her recovery status is unknown.

The cause of her infarctive stroke is unclear but can be inferred to be due to the traumatic brain injury both from the ball and frequent neck massage. The major limitation of this study is the limited data on the cause and effect of blunt trauma from neck massagers and the unavailable object assessment of it resulting in stroke. Clinicians ought to be vigilant regarding stroke cases of unknown origin, prompting thorough investigations into any history of past trauma, whether repetitive or singular. More research is recommended to investigate the direct cause of such blunt trauma in causing strokes. The widespread adoption of devices such as neck massagers among the general public warrants attention, as they could potentially induce repetitive vessel trauma. Therefore, caution in the use of such devices is encouraged.
